# The influence of nitrogen availability on anatomical and physiological responses of *Populus alba × P. glandulosa* to drought stress

**DOI:** 10.1186/s12870-019-1667-4

**Published:** 2019-02-08

**Authors:** Junyu Song, Yang Wang, Yuehan Pan, Jiayin Pang, Xin Zhang, Junfeng Fan, Yi Zhang

**Affiliations:** 1 State Key Laboratory of Crop Stress Biology in Arid Areas, College of Forestry, Northwest A&F University, Yangling, 712100 Shaanxi China; 2 The UWA Institute of Agriculture, and the School of Agriculture and Environment, The University of Western Australia, Perth, WA 6001 Australia; 3 School of Biological Science, The University of Western Australia, Perth, WA 6001 Australia

**Keywords:** Drought tolerance, Nitrogen, *Populus*, Phytohormone, Antioxidant defense

## Abstract

**Background:**

Drought and nitrogen (N) deficiency are two major limiting factors for forest productivity in many ecosystems. Elucidating the mechanisms underlying the influence of soil N availability on drought responses of tree species is crucial to improve tree growth under drought.

**Results:**

The root proliferation under drought was enhanced by adequate N application. Vessel frequency in xylem increased upon drought, with more significant increase under adequate N conditions compared with that under low N conditions, possibly leading to increased hydraulic safety. Nitrogen application under drought increased indole acetic acid (IAA), which contributed to the adaptive changes of xylem. Nitrogen application increased leaf abscisic acid (ABA) concentration, therefore regulated stomata adjustment, and promoted intrinsic water use efficiency (*WUE*_*i*_). Moreover, N application promoted antioxidant defense in leaves by showing increased level of free proline and carotenoid, which improved drought tolerance and growth performance of poplars.

**Conclusions:**

Anatomical and physiological responses of *Populus* to drought were suppressed by N deficiency. Adequate N application promoted adaptive changes of root and xylem under drought and increased hydraulic safety. Nitrogen addition under drought also increased leaf ABA level which may regulate stomata adjustment and promote *WUE*_*i*_. Moreover, nitrogen application improved antioxidant defense in leaves with increased levels of antioxidants. These positive regulations improved drought tolerance and growth performance of poplars.

**Electronic supplementary material:**

The online version of this article (10.1186/s12870-019-1667-4) contains supplementary material, which is available to authorized users.

## Background

Drought is a major inhibiting factor for plant growth and productivity in many ecosystems [1–3]. Drought may reduce forest productivity and even lead to increased forest mortality around the world [4]. The global climate change leads to more frequent drought events in the semiarid or semi-humid areas [4, 5]. Therefore, it is crucial to elucidate the mechanism of drought acclimation in tree species.

Plants can cope with drought through drought avoidance and drought tolerance [4, 6]. Drought avoidance involves stomata adjustment which may reduce leaf transpiration rate and increase intrinsic water use efficiency (*WUE*_*i*_) [7–9]. Plants also exhibit anatomical changes in stems and roots which can reduce hydraulic conductivity and leaf water losses [2]. Drought tolerance mainly involves production of antioxidants and osmolytes such as soluble sugars, which may facilitate the homeostasis of reactive oxygen species (ROS) and benefit cell and tissue activity under water deficit [4, 6, 10–12]. Moreover, drought can alter root architecture and induce root phenotypic plasticity [11]. For example, fine roots can be inhibited under drought in line with the drought-tolerance strategy, resulting in a decrease in specific root length (SRL) [11].

In addition to water deficit, nutrient deficiency is another constraint to plant growth [13–16]. Forest plantations are often located in marginal lands with limited water and nutrient resources due to the increasing demand in agriculture products and limited farmland area [10, 17]. Therefore, the interactive effects of co-occurring nutrient and water conditions are crucial for forest ecosystems [18]. During drought stress, adequate nutrient availability can increases water use efficiency, minimizes negative effect of drought and promotes faster recovery after drought [18, 19]. Elucidating the underlying mechanisms by which nutrient availability alleviates drought stress will be crucial for tree growth and forest productivity [18]. However, current research is mainly focused on the water- and carbon-associated mechanisms underlying drought tolerance. The interaction between nutrients and drought tolerance of plants are still not well addressed.

Nitrogen (N) is critical for plants to produce metabolites and components in various biological processes such as amino acids, protein, nucleotides, and chlorophyll [15, 20]. Nitrogen application might promote the physiological responses to drought via elevating N and chlorophyll concentrations, and enhancing PSII photochemical activity [17]. The positive relationship between water use efficiency (WUE) and N addition has been demonstrated, and N might promoted WUE via stimulating plant dry mass and reducing water loss [16]. Moreover, nitrogen may alleviate the inhibitory effects of drought on photosynthesis and avoid C starvation [18, 19]. Low N availability can increase sensitivity upon drought and triggers protein degradation, leading to decrement of N-containing osmolytes such as proline [10, 18].

*Populus* is an important woody crop for pulp industry and bioenergy, and it is widely distributed in the semiarid area of China, where it has an annual precipitation of 500~700 mm and rainfall often occurs intermittently, leading to frequent short-term drought [21]. Moreover, *Populu*s trees are often planted on marginal lands with limited water and nutrient resource [8, 22, 23]. Previous studies on *Populus* species have demonstrated the mechanisms underlying drought stress [1, 6, 8] without the consideration of N effect. Under drought, *Populus* species demonstrated suppressed growth, increased ABA level, altered anatomical properties and increased δ^13^C and *WUE*_*i*_ [8, 12]. *Populus* also developed xylem with narrower vessel lumen and higher vessel frequency under drought stress, which may facilitate hydraulic safety and avoid embolism [2, 8].

In this study, an integrative investigation on root parameters, anatomical properties of xylem and physiological responses such as the induction of phytohormones and antioxidants were conducted under combined conditions of drought and two contrasting N levels (adequate-N and low-N). The objective of this study was to elucidate the mechanisms underlying the influence of N availability on adaptive responses of *Populus* under drought stress.

## Results

### The impact of water status and N conditions on growth traits, root parameters and N uptake

**Fig. 1:**
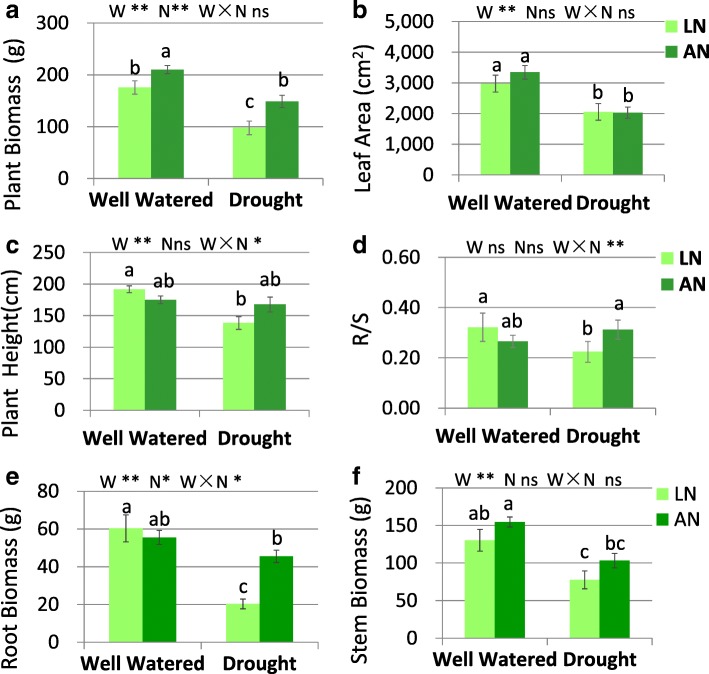
Biomass (**a**), R/S (**b**), tree height (**c**), leaf area (**d**), root biomass (**e**) and stem biomass (**f**) under drought and well-watered conditions with low nitrogen (LN) or adequate nitrogen (AN). R/S, the ratio of root biomass to shoot biomass. The bars indicate means ± SE (*n* = 12). Different letters on the bars indicate significant differences (*P* < 0.05) based on multiple comparisons (Tukey’s HSD test) in ANOVA. *P*-values of the two-way ANOVA of water (W), nitrogen (N) and their interactions (W × N) are indicated. *P < 0.05; **P < 0.01; ns, not significant

Water treatment had significant effect on plant biomass (Fig. 1a) and leaf area (Fig. 1b). Nitrogen treatment had significant effect on plant biomass. Drought significantly reduced plant biomass (Fig. 1a, *P* < 0.01), leaf area (Fig. 1b, *P* < 0.01) and stem biomass (Fig. 1f, *P* < 0.01) under both low N and adequate N condition. Nitrogen application increased plant biomass under both well-watered and drought conditions. For plant height (Fig. 1c), R/S (Fig. 1d) and root biomass (Fig. 1e), significant interactions of water × N were found. Drought stress reduced plant height, R/S and root biomass under low N condition, but not under adequate-N supply (Fig. 1). Under drought, N application dramatically increased root biomass (*P* < 0.05).

**Fig. 2:**
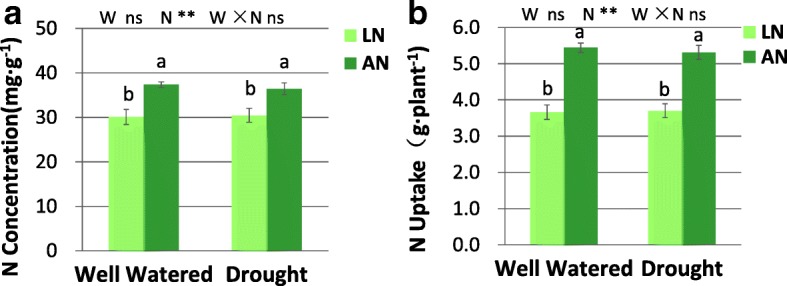
Leaf N concentration (**a**) and N uptake (**b**) under drought and well-watered conditions with low nitrogen (LN) or adequate nitrogen (AN). The bars indicate means ± SE (*n* = 12). Different letters on the bars indicate significant differences (*P* < 0.05) based on multiple comparisons (Tukey’s HSD test) in ANOVA. *P*-values of the two-way ANOVA of water (W), nitrogen (N) and their interactions (W × N) are indicated. *P < 0.05; **P < 0.01; ns, not significant

Two-way ANOVA showed interaction of water × N for all root morphological parameters (Table 1). Multiple comparisons indicated that when N supply was low, root parameters including root length, root surface area, number of root tips, fine root length and fine root surface area all were decreased by drought stress (Table 1). In contrast, root morphological parameters were not affected by drought when adequate N was supplied (Table 1). Under drought condition, N application increased most root parameters. In contrast, root parameters were not altered by N application in well-watered treatment (Table 1). Leaf N concentration and the total amount of N uptake by plants were increased by N application, while not altered by water status under both N conditions (Fig. 2).

**Table 1:** Root parameters under each combination of water and nitrogen condition

Nitrogen	Water	Root Length (cm)	Root SurfArea (cm^2^)	Number of Root Tips	Fine Root Length (cm)	Fine Root SurfArea (cm^2^)
Well Watered	Low Nitrogen	1311.7 a ±155.0	385.5 a ±43.2	1056.4 a ±838.4	672.8 a ±97.9	23.9 a ±3.4
	Adequate Nitrogen	1190.3 a ±95.5	388.6 a ±72.1	877.6 ab ±451.7	564.2 a ±53.2	20.1 a ±1.9
Drought	Low Nitrogen	674.1 b ±198.3	191.0 b ±60.8	535.4 b ±243.5	313.2 b ±94.2	11.2 b ±3.4
	Adequat Nitrogen	1422.3 a ±147.8	431.1 a ±48.3	1174.8 a ±572.8	735.5 a ±84.7	26.2 a ±3.1
*F* Value of Two-way ANOVA	Nitrogen (N)	4.3*	4.7*	3.5	3.6	3.6
	Water (W)	1.8	1.8	0.8	1.3	1.3
	N × W	8.4**	4.5*	10.9**	10.3**	10.4**

SurfArea: root surface area. Means with different letters are statistically different in multiple comparisons between treatments at the 5% level in ANOVAs. Level of significance of *F* value in two-way ANOVA is denoted by: *0.05 > *p* > 0.01, **p < 0.01

### The impact of drought and N status on gas exchange parameters

**Fig. 3:**
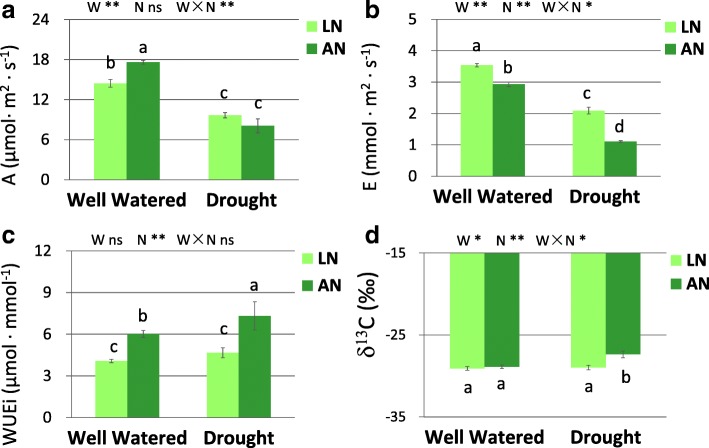
Average value of *A* (**a**), *E* (**b**), *WUE*_*i*_ (**c**) and δ^13^C (**d**) in leaves. LN, low nitrogen. AN, adequate nitrogen. *A*, net photosynthetic rates. *E*, transpiration rates. *WUE*_*i*_, intrinsic water use efficiency. δ^13^C, stable carbon isotope compositions. The bars indicate means ± SE (*n* = 12). Different letters on the bars indicate significant differences (*P* < 0.05) based on multiple comparisons (Tukey’s HSD test) in ANOVA. *P*-values of the two-way ANOVA of water (W), nitrogen (N) and their interactions (W × N) are indicated. *P < 0.05; **P < 0.01; ns, not significant

N treatment had significant effect on *WUE*_*i*_, and there was an interaction of water × nitrogen for *A*, *E* and δ^13^C (Fig. 3). Drought reduced *A* and *E* under both low- and adequate- N conditions, with more decrease under adequate-N than that under low-N (Fig. 3a, b). *A* was increased by N application only under well-watered condition. *E* was decreased by N application under both water conditions, with more decrease under drought than that under well-watered condition. *WUE*_*i*_ was affected by N levels, with *WUE*_*i*_ being significantly higher with the addition of N (Fig. 3c). N application increased δ^13^C under drought, but not under well-watered condition (Fig. 3d).

### The impact of drought on anatomical features of stems as affected by N availability

**Fig. 4:**
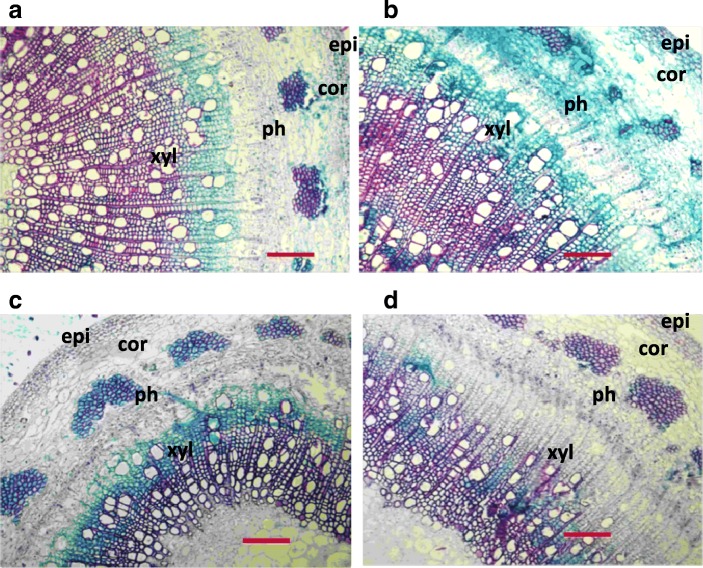
Anatomical properties of stem under each combined treatment of water and nitrogen. **a** adequate nitrogen and drought; **b** adequate nitrogen and well-watered; **c** low nitrogen and drought; **d** low nitrogen and well-watered. Epi, epidermis; cor, cortex; ph, pholem; xyl, xylem; Bar = 200 μm

Both N and water treatment had significant effect on the number of xylem cell layers. There were interactive effects of water × nitrogen for vessel frequency, xylem thickness and fiber lumen diameter (Table 2). Vessel frequency increased upon drought with more increase under adequate-N condition (91%) compared with that under low-N condition (42%) (Table 2). Xylem thickness decreased upon drought with larger reduction under low-N condition compared with that under adequate-N condition (Fig. 4; Table 2). Vessel lumen diameter did not change upon drought under both N conditions. Nitrogen deficiency decreased vessel frequency only in drought treatment. Nitrogen deficiency decreased fiber lumen diameter only under well-watered condition. N deficiency decreased xylem thickness and the number of xylem cell layers under both water conditions (Table 2).

**Table 2:** Stem anatomical properties under each combination of water and nitrogen condition

Nitrogen Conditions	Water Conditions	VF (number mm^−2^)	VLD (μm)	FLD (μm)	XT (mm)	XCL	PT (mm)
Well Watered	Low Nitrogen	68.8 c±4.6	45.4 a ±2.5	13.5 b ±0.3	0.51 b ±0.02	37.9 b ±0.9	0.10 b ±0.01
	Adequate Nitrogen	72.9 c ±13.0	45.6 a ±2.5	15.7 a ±0.3	0.77 a ±0.03	49.3 a ±2.2	0.11 ab ±0.01
Drought	Low Nitrogen	97.8 b ±3.9	45.2 a ±2.3	13.2 b ±0.6	0.34 c ±0.03	26.1 c ±1.9	0.11 ab ±0.01
	Adequate Nitrogen	139.6 a ±9.5	39.5 a ±5.5	12.7 b ±0.7	0.5 b ±0.02	39.6 b ±2.3	0.12 a ±0.01
*F* Value of Two-way ANOVA	Nitrogen (N)	10.7*	1.9	8.3*	345.1**	133.2**	5.8*
	Water (W)	49.6**	2.4	31.9**	378.8**	105.6**	1.4
	N × W	8.8*	2.2	20.7**	19.6**	1.2	0.00

VF vessel frequency; VLD vessel lumen diameter; FLD fiber lumen diameter; XT xylem thickness; XCL xylem cell layers; PT phloem thickness. Means with different letters are statistically different in multiple comparisons between treatments at the 5% level in ANOVAs. Level of significance of *F* value in two-way ANOVA is denoted by: *0.05 > *p* > 0.01, **p < 0.01

### Physiological responses to drought as affected by N conditions

**Fig. 5:**
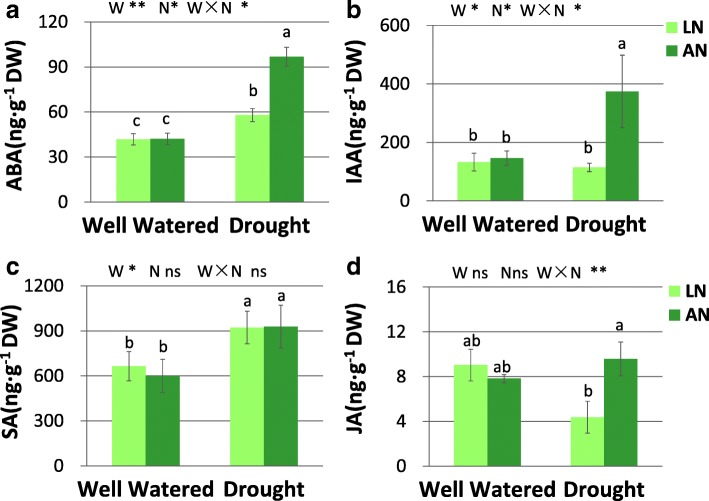
Concentration of ABA (**a**), IAA (**b**), SA (**c**) and JA (**d**) in leaves. LN, low nitrogen. AN, adequate nitrogen. ABA, abscisic acid. IAA, indole acetic acid. SA, salicylic acid. JA, jasmonic acid. The bars indicate means ± SE (*n* = 4). Different letters on the bars indicate significant differences (*P* < 0.05) based on multiple comparisons (Tukey’s HSD test) in ANOVA. *P*-values of the two-way ANOVA of water (W), nitrogen (N) and their interactions (W × N) are indicated. *P < 0.05; **P < 0.01; ns, not significant

Two-way ANOVA showed the interaction of water × nitrogen for ABA*,* IAA and JA levels. The water treatment had significant effect on SA level (Fig. 5). In response to drought, ABA concentration increased under both low- and adequate- N conditions, with more increase under adequate-N (by 109%) than that under low-N (by 39%). (Fig. 5a). IAA level increased upon drought only under adequate N condition (Fig. 5b). SA concentration was increased by drought under both N conditions (Fig. 5c). (Fig. 5c). JA level increased in response to N application only under drought, and was not altered by drought regardless of N conditions, (Fig. 5d).

**Fig. 6:**
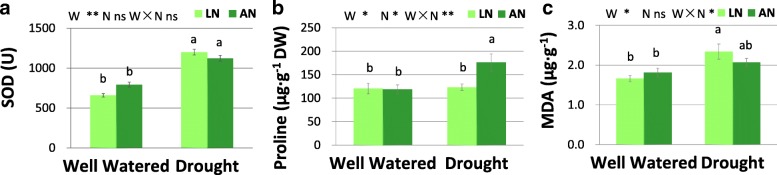
The levels of SOD (**a**), free proline (**b**) and MDA (**c**) in leaves. SOD, superoxide dismutase. MDA, malonaldehyde. LN, low nitrogen. AN, adequate nitrogen. The bars indicate means ± SE (*n* = 12). Different letters on the bars indicate significant differences (*P* < 0.05) based on multiple comparisons (Tukey’s HSD test) in ANOVA. *P*-values of the two-way ANOVA of water (W), nitrogen (N) and their interactions (W × N) are indicated. *P < 0.05; **P < 0.01; ns, not significant

**Fig. 7:**
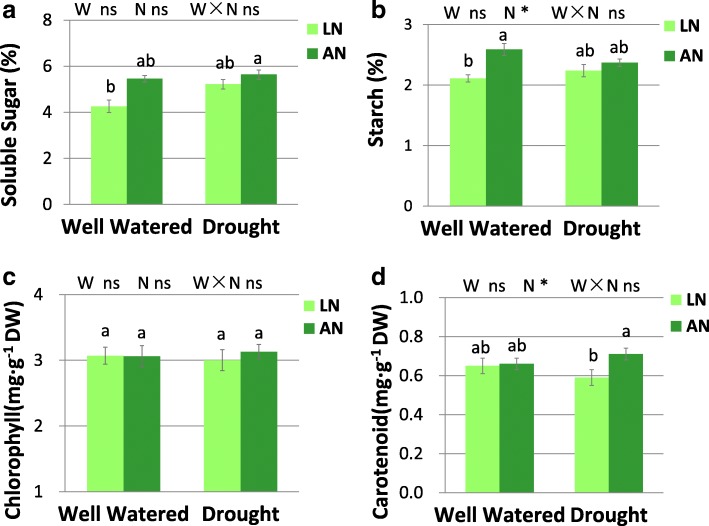
Concentration of soluble sugar (**a**), starch (**b**), chlorophyll (**c**) and carotenoid (**d**) in leaves. LN, low nitrogen. AN, adequate nitrogen. The bars indicate means ± SE (*n* = 12). Different letters on the bars indicate significant differences (*P* < 0.05) based on multiple comparisons (Tukey’s HSD test) in ANOVA. *P*-values of the two-way ANOVA of water (W), nitrogen (N) and their interactions (W × N) are indicated. *P < 0.05; **P < 0.01; ns, not significant

Water treatment had significant effect on SOD activity (Fig. 6a). Significant interaction of water × nitrogen was found for proline (Fig. 6b) and MDA (Fig. 6c) levels (Fig. 6). SOD activity was increased by drought regardless of N conditions. Free proline concentration was elevated upon drought only under adequate N condition. Drought increased MDA concentration under low-N but not under adequate-N condition (Fig. 6). The level of free proline increased upon N addition under drought, but not under well-watered condition (Fig. 6). The concentration of chlorophyll and carotenoid were not altered by water treatment. N application increased carotenoid concentration only under drought condition (Fig. 7). N addition increased starch concentration only under well-watered condition (Fig. 7).

## Discussion

### Plant growth and root traits under drought stress are influenced by N availability

Plant growth and root traits were inhibited by drought regardless of N conditions, but the inhibition was more significant under low-N conditions. It has been widely reported that root traits are tightly associated with plant productivity under drought [4, 24, 25], and fertilizer application may mitigate the negative effects of water deficit by improving root growth and water use efficiency [22, 26, 27]. Nitrogen application may stimulate root growth and increase root plasticity under drought, which contribute to the maintenance of growth and dry matter production [27]. Under drought condition in the present study, root parameters including root length, root surface area, number of root tips, fine root length and fine root surface area were all increased by adequate N addition, indicating that appropriate N nutrition can alleviate the inhibitive effects of drought stress on root growth and thus facilitate seedling growth and dry matter production of poplar.

### Stem anatomical changes under drought as affected by N availability

Anatomical analysis showed that xylem development were suppressed by drought, while the suppression was partially alleviated by N application, presumably due to the role of nitrogen in the secondary cell wall formation in xylem [28–31]. In acclimation to long-term drought, tree species including *Populus* usually produce wood with more numerous and narrower vessels, which may increase hydraulic safety and reduce the risk of embolism and die-back under drought [2, 8]. In this study, vessel frequency increased upon drought regardless of N conditions, while the extent of increment was greater under adequate N condition than under low N condition, which can promote hydraulic safety and reduce the risk of dieback resulting from embolism, as reported previously [30]. Compared with low vessel frequency, high vessel frequency can increase hydraulic safety as a larger number of vessels will stay functional at a certain rate of xylem embolism [32]. However, a high vessel frequency can also increase cavitation probability as embolisms spread may be more intense. In stressful conditions, the effects of vessel frequency on hydraulic safety and cavitation spread can be balanced in acclimation to drought [32]. In contrast with previous reports [2, 8], the vessel lumen diameter did not decrease under drought in the present study. This may be explained as the genotype-specific response of xylem anatomy to drought and/or the differences in the duration of drought treatment [30]. Some deciduous tree species may exhibit unaltered hydraulic conductivity and vessel size under drought, but they can be exempted from the embolism as they shed leaves during drought, which may reduce the high pressure within a vessel [30]. Further investigations are needed to elucidate whether this strategy of drought adaptation is also applicable in poplar.

### The influence of N availability on gas exchanges and hormone signals

In the present study, gas exchange characteristics including *A* and *E* decreased upon drought stress regardless of N conditions. These adaptive changes can reduce water loss and facilitate the acclimation of plants to drought [7, 8]. Sufficient N may promote water use efficiency, while N deficiency may impair the ability of the plants to regulate stomata movement according to soil moisture [18, 33, 34]. In the present study, appropriate N application changed gas exchange characteristics and increased *WUE*_*i*_ under both water conditions, while the causes and the implication of these changes were different under different watering regimes. Under well-watered condition, N application decreased *E* and increased *A* and *WUE*_*i*_, which may facilitate the production of carbohydrate and provide basic compounds for rapid biomass production together with the increased N assimilation and protein biosynthesis [22]. Under drought stress, *E* was decreased by N application, while *A* remained unaltered. As a result, *WUE*_*i*_ were higher under adequate N condition than under N deficiency, indicating that appropriate N application may be beneficial for the regulation of gas exchange and improving water use efficiency of poplars under drought stress.

Further analysis revealed that ABA level was induced by drought, with more significant increase under adequate-N condition compared with that under low N condition. The level of ABA signal are usually induced by water deficit, which may regulate the stomata movement and alleviate water losses under drought stress [11, 12]. Previous study found that ABA concentration may be higher in N-fertilized plants than non-fertilized plants [10]. The present study demonstrated that N application contributed to the sensitive stomata adjustment and high *WUE*_*i*_ under drought via enhancing the inducement of ABA upon drought. In addition to ABA, JA and IAA also participate in abiotic stress responses through interaction with ABA in a complex manner [7, 9, 35]. The transcription factors AtMYC2 has been shown to be the convergent points of JA/ABA signals [35]. Similarly, auxin also has synergistic effect on ABA signaling, while ABA can enhance auxin signaling by activating auxin-responsive promoters [36]. In this study, the biosynthesis of ABA, JA, and IAA were synergistically induced upon drought when soil N was appropriate, while the inducement was absent under low-N condition. These results indicated that the synergistic inducement of ABA, JA, and IAA under drought were related to N status, and the induced phytohormones under adequate N condition may cooperatively participated in drought tolerance.

As a key growth regulating phytohormone, auxin can activate cell division and enlargement in leaf and stem [36–39]. Elevated auxin concentration may promote vessel differentiation and increase the frequency of vessel differentiation in xylem [36]. IAA usually moves from the source tissues (young leaves and flowers) to stem via a bulk flow in the mature phloem [40]. Therefore, the elevated IAA concentration in leaves can also influence the IAA level in stem, which may regulate the cell division and proliferation in the xylem, resulting in an increment of vessel frequency. Moreover, auxin can simulate xylem development via increasing xylem thickness and the number of xylem cells. Therefore, the elevated IAA concentration might have contributed to the N-simulated xylem development in the poplar clone.

### The influence of N availability on antioxidant defense of leaves under drought stress

In response to drought stress, plants usually accumulate osmolytes such as amino acids and soluble sugars to reduce the osmotic potential [8, 41]. After sensing the signal of water deficit, plants adjust the metabolism of C and N, and maintain the homeostasis of ROS production and scavenging [8]. Although short-term drought may activate the production of osmolytes such as soluble sugars and sugar alcohol, long-term drought may inhibit carbohydrates production as a result of decreased CO_2_ assimilation [42]. In this study, the level of soluble sugar remain stable during drought despite of the decreased photosynthesis rates and carbon assimilation under drought. The stable level of soluble sugar may be maintained at the expense of starch and inversion of some carbonhydrates [8].

Plants usually activate antioxidant enzymes and non-enzymatic antioxidants such as free proline and carotenoid to maintain the homeostasis of ROS production and scavenging [42–44]. In this study, SOD activity increased in response to drought under both N conditions, while it was unaffected by N treatment, indicating that SOD may be crucial for scavenging ROS and protecting tissues from oxidative damage under both adequate and low soil N conditions. Proline is involved in osmotic adjustment, preservation of enzyme structure and activity, and protection of membrane under stress [8, 45]. Carotenoid concentration and Car/Chl ratio are involved in the light protecting mechanisms, and participate in antioxidant defense by inhibiting lipid peroxidation and stabilizing membranes [41, 43]. We demonstrated that adequate N application promoted the levels of free proline and carotenoid, which may promote drought tolerance via promoting antioxidant defense and membrane protection. Proline accounted for 25–85% of the free amino acid accumulation in leaves [45]. The promotion of free proline by N application may be due to a better capacity for amino acid synthesis in adequate N-supplied plants [45]. Leaf malondialdehyde (MDA) concentration, an important indicator for oxidative damages under stress [4, 8], was increased by drought when soil N was deficient, while it remain stable upon drought in N-sufficient treatment. This result indicated that leaves are better protected against the adverse effects of drought stress due to the improved antioxidative defense under adequate N condition.

## Conclusions

**Fig. 8:**
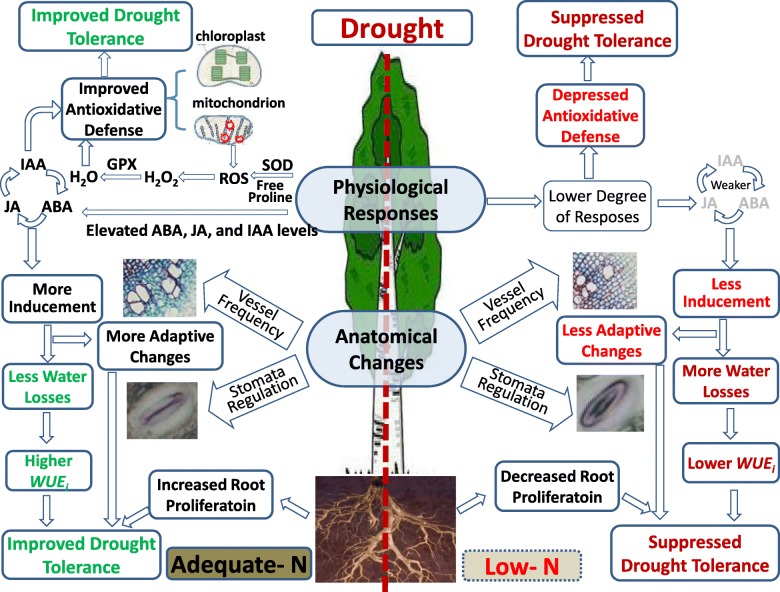
The schematic model of drought tolerance of *Populus* as affected by adequate- and low- N condition. The root proliferation under drought was suppressed by N deficiency and enhanced by adequate N application, which may benefit water uptake under drought. The degree of adaptive changes of xylem and the increment of vessel frequency under drought were enhanced by adequate-N application, which may reduce the water losses. The elevated IAA after N application contributed to the adaptive changes of xylem under drought, and promoted the secondary development of stem. N application increased the concentration of ABA in leaves, which contributed to the sensitive stomata adjustment and promoted *WUE*_*i*_ and long-term water use efficiency under drought. The biosynthesis of ABA, IAA and JA were synergistically promoted by N application and they cooperatively participated in drought tolerance. Moreover, N application improved antioxidant defense in leaves via regulating the production of N-related antioxidants including proline and carotenoid, and thus lead to improved drought tolerance and better growth performance of poplars

Mechanisms underlying the influence of soil N availability on drought acclimation of *Populus* were demonstrated, which could be summarized as a schematic model (Fig. 8). Root proliferation under drought was enhanced by adequate N application, which may be crucial for drought acclimation and plant growth. The degree of adaptive changes of xylem upon drought were enhanced by adequate-N application, attributing to an increased vessel frequency and promoted hydraulic safety under drought. The elevated IAA under adequate N condition contributed to the adaptive changes of xylem and promoted the secondary development of stem. Adequate N application increased the concentration of ABA in leaves, which contributed to sensitive stomata adjustment and increased *WUE*_*i*_ under drought. The increased ABA, IAA and JA under adequate N condition may cooperatively contribute to drought tolerance. Moreover, adequate N supply improved antioxidant defense in leaves via regulating the production of N-related antioxidants including proline and carotenoid, and thus lead to better drought tolerance and growth performance. According to these results, adequate N fertilization and/or silviculture strategies with positive effects on soil N fertility are proposed in poplar plantations that are limited by both water and nitrogen, with the purpose to improve drought adaptation and growth performance of poplars.

## Methods

### Plant material and experimental design

A poplar clone of *Populus alba × P. glandulosa* that is widely distributed and well-adapted in North China was used for this study. Seedlings were purchased from the Research Institute of Forestry, Chinese Academy of Forestry. Experiments were undertaken in Northwest A&F University, China.

The rooted plantlets in similar size were transferred to plastic pots filled with 10 L of the substrate, and subsequently cultivated in a growth chamber (day/night temperature, 28/20 °C; relative air humidity, 50–60%; photoperiod per day, 14 h; light intensity, 200 μmol m^− 2^ s^− 1^). The substrate was the mixture of sand and soil (sand: soil, 1:1, *v*/v), and the soil type was loess, a typical soil type in northwest China with low level of available nutrients including N. The mixed substrate had a pH of 7.9, and the available N, P and K were 8, 3 and 11 mg·kg^− 1^, respectively. The experiment was started in March 2016. After the transplanting of seedlings, 50 ml Long Ashton (LA) nutrient solution was supplied to each pot every 3 days. The LA solution contains 1 mM NH_4_NO_3_, 0.5 mM KCl, 0.9 mM CaCl_2_, 0.3 mM MgSO_4_, 0.6 mM KH_2_PO_4_, 42 μM K_2_HPO_4_, 10 μM Fe-EDTA, 2 μM MnSO_4_, 10 μM H_3_BO_3_, 7 μM Na_2_MoO_4_, 0.05 μM CoSO_4_, 0.2 μM ZnSO_4_, and 0.2 μM CuSO_4_. After 6 weeks of transplanting, plants in similar size (c. 50 cm in height) were selected for the study on the interactive effects of N and watering treatments.

This study used a two-factorial design consisting of two N levels (adequate-N and low-N) and two watering treatments (drought and well-watered), giving a combination of four treatments with 12 replicates in each treatment. The N treatments were started on 10th April, 2016 and lasted for 50 days until harvest. Twenty days after the N treatments, two watering treatments were imposed in combination with the N treatments, which were lasted for 30 days until the final harvest. The composition of the LA nutrient solution was the same as that mentioned above except NH_4_NO_3_, which was 100 μM or 1000 μM for the low-N and adequate-N treatment, respectively. The LA nutrient solution was supplied at a rate of 100 mL per pot every 3 days. In the well-watered treatment, each pot were kept well-watered at ~ 70% field capacity by weighing and watering every two days. In the drought-stressed treatment, each pot had watering withheld initially, when the soil water content dropped to ~ 40% field capacity, it was maintained at that level by weighing and watering back every two days.

### Gas exchange measurement

Just prior to the harvest, three leaves of each sapling (leaf plastochron index = 8–10) were used to measure net photosynthetic rates (*A*), transpiration rates (*E*) using a portable photosynthesis system (Yaxin-1102), with a LED light source [4]. The measurements were conducted between 09:00 h to 11:00 h, and the light intensity was 1000 μmol · m^2^ · s^− 1^, the CO_2_ concentration was 400 μmol·mol^− 1^. The *WUE*_*i*_, the ratio of *A* to *E* were determined. After the measurements of gas exchange, the same group of leaves were used for microscopic analysis, physiological measurement and transcriptome analysis.

### Harvest procedure

At harvest, plants were separated into leaves, stems and roots after plant height was measured. For the analysis of physiological parameters, leaf samples from 12 seedlings in each treatment were collected and were frozen in liquid N_2_, and then samples were stored in a freezer at − 80 °C. The fifth internodes of stems (at the location of *approx.* 10 cm below the apex of stem) were collected for anatomical analysis. Root morphological parameters were measured immediately after harvest using a Win/MacRHIZO root analysis system (Régent Instruments, Quebec, Canada). Shoots and roots were dried at 70 °C for 72 h, and dry weight (DW) was measured. The ratio of root DW to the shoot DW (R/S) was calculated. Nitrogen concentration was analyzed using an auto-analyzer (Kjeltec 2300 Analyzer Unit, Foss, Sweden). The N content was determined by multiplying N concentration of each organ with dry weight of that organ.

### Microscopic analysis of anatomical features of stem

Anatomical properties of stem cross section were analyzed according to previous method [12]. Transverse cross sections (10 μm thick) were obtained with the sliding microtome and stained with toluidine blue. Stained sections were photographed and analyzed. The diameter of vessel lumen, vessel frequency (the number of vessels per unit area on the cross section) and vessel lumen area were quantified as previously described [8].

### Measurement of soluble sugars, starch, free proline, phytohormones and antioxidant enzymes

In the analysis of physiological parameters, leaf samples from 12 seedlings in each treatment were ground to fine powder under liquid N_2_. Soluble sugar was analyzed as previously described [10]. Starch content and free proline were measured using the method of Chołuj et al. [46]. Concentrations of chlorophyll and carotenoid in leaves were determined according to the method of Zheng et al. [4]. Considering the tremendous quantity of work in the measurement of phytohormones, samples from three plants in each treatment were combined to form a mixed sample, which produced four statistical replicates from the 12 seedlings in each treatment. The levels of hormones including indole acetic acid (IAA), salicylic acid (SA), jasmonic acid (JA), and abscisic acid (ABA) were determined using a high-performance liquid chromatography-electrospray ionization-ion trap mass spectrometry (HPLC-MS) [13].

The activity of superoxide dismutase (SOD) (EC 1.15.1.1) was determined according to the method of Cao et al [8]. One unit of SOD was defined as the amount of enzyme that catalyze 50% SOD-inhibited nitroblue tetrazolium at 550 nm. The malonaldehyde (MDA) concentrations were analyzed spectrophotometrically at the wavelength of 450, 532, and 600 nm as described previously [8].

### Determination of total C and stable carbon isotope compositions

The determination of total C and stable carbon isotope compositions (δ^13^C) followed the method by Zheng et al [4]. The ratio of ^13^C to ^12^C is denoted as parts per thousand deviations (‰) from the Pee Dee Belemnite (PDB) standard [12]. Carbon isotope composition was calculated as:

$$ {\updelta}^{13}\mathrm{C}=\left({\mathrm{R}}_{\mathrm{sa}}-{\mathrm{R}}_{\mathrm{sd}}\right)/\left({\mathrm{R}}_{\mathrm{sd}}\times 1000\right)\left({\mbox{\fontencoding{U}\fontfamily{wasy}\selectfont\char104}} \right) $$

where R_sa_ and R_sd_ are the ratios of ^13^C to ^12^C of the sample and the standard, respectively. Nitrogen content was measured by an auto-analyzer (Kjeltec 2300 Analyzer Unit, Foss, Sweden). The total N amount in each organ was determined by multiplying the dry weight of each organ with N concentration of that organ.

### Statistical analysis

The UNIVARIATE procedure in SAS software (SAS Institute, Cary, NC; 1996) was used to test the normality of all data. To examine the effects of nitrogen and watering treatment, all variables were analyzed by two-way ANOVA using SAS software, and *P*-values of the ANOVA are indicated. To investigate the differences in variables between the four treatments (two nitrogen levels by two water levels), multiple comparisons (Tukey’s HSD test) were conducted, and the means with different letters indicate significant differences at *P* = 0.05. The homogeneity of variances was tested using the method of LEVENE (SAS Institute, Cary, NC; 1996) (Additional file 1: Table S1).

## Additional file

Additional file 1: **Table S1.** Levene’s test for homogeneity of variance in ANOVA. (DOCX 41 kb)

## Abbreviations

*A*: Net photosynthetic rates
ABA: Abscisic acid
ABA: Abscisic acid
*E*: Transpiration rates
IAA: Indole acetic acid
JA: Jasmonic acid
MDA: Malonaldehyde
ROS: Reactive oxygen species
SA: Salicylic acid
SOD: Superoxide dismutase
*WUE*_*i*_: Intrinsic water use efficiency
δ^13^C: Stable carbon isotope compositions
