# Supplementary material for: The influence of nitrogen availability on anatomical and physiological responses of Populus alba × P. glandulosa to drought stress
Source: BMC Plant Biol. 2019 Feb 8;19:63. doi: 10.1186/s12870-019-1667-4 (PMC6368793; doi:10.1186/s12870-019-1667-4)
Supplement: Supplementary file 1 — Table S1. Levene’s test for homogeneity of variance in ANOVA. (DOCX 41 kb) [file 12870_2019_1667_MOESM1_ESM.docx]

| Table S1 Levene's test for homogeneity of variance in ANOVA | | | | | |
| --- | --- | --- | --- | --- | --- |
| Variables | | Degree of Freedom | F Value | *Pr* > F |  |
| Variables in Figure 1 | Biomass | 3 | 0.42 | 0.74 |  |
|  | R/S | 3 | 0.96 | 0.43 |  |
|  | Tree Height | 3 | 2.09 | 0.13 |  |
|  | Leaf Area | 3 | 0.57 | 0.64 |  |
|  |  |  |  |  |  |
| Variables in Figure 2 | N Concentration | 3 | 2.49 | 0.07 |  |
|  | N Uptake | 3 | 0.53 | 0.66 |  |
|  |  |  |  |  |  |
| Variables in Figure 3 | *A* | 3 | 1.64 | 0.23 |  |
|  | *E* | 3 | 1.44 | 0.28 |  |
|  | *WUE_i_* | 3 | 2.49 | 0.11 |  |
|  | δ^13^C | 3 | 1.09 | 0.39 |  |
|  |  |  |  |  |  |
| Variables in Figure 5 | ABA | 3 | 1.51 | 0.26 |  |
|  | IAA | 3 | 2.76 | 0.08 |  |
|  | SA | 3 | 1.59 | 0.24 |  |
|  | JA | 3 | 1.23 | 0.34 |  |
|  |  |  |  |  |  |
| Variables in Figure 6 | SOD | 3 | 1.60 | 0.24 |  |
|  | Proline | 3 | 2.45 | 0.07 |  |
|  | MDA | 3 | 2.00 | 0.12 |  |
|  |  |  |  |  |  |
| Variables in Figure 7 | Soluble Sugars | 3 | 1.83 | 0.15 |  |
|  | Starch | 3 | 1.09 | 0.36 |  |
|  | Chlorophyll | 3 | 0.78 | 0.51 |  |
|  | Carotenoid | 3 | 0.96 | 0.42 |  |
